# Deep Convolutional Neural Network-Based Epileptic Electroencephalogram (EEG) Signal Classification

**DOI:** 10.3389/fneur.2020.00375

**Published:** 2020-05-22

**Authors:** Yunyuan Gao, Bo Gao, Qiang Chen, Jia Liu, Yingchun Zhang

**Affiliations:** ^1^School of Automation, Intelligent Control and Robotics Institute, College of Automation, Hangzhou Dianzi University, Hangzhou, China; ^2^Key Laboratory of Brain Machine Collaborative Intelligence of Zhejiang Province, Hangzhou, China; ^3^Department of Industrial and Systems Engineering, Auburn University, Auburn, AL, United States; ^4^Department of Biomedical Engineering, University of Houston, Houston, TX, United States

**Keywords:** epileptic EEG signal classification, power spectrum density energy diagram, deep convolutional neural networks, electroencephalogram, EEG

## Abstract

Electroencephalogram (EEG) signals contain vital information on the electrical activities of the brain and are widely used to aid epilepsy analysis. A challenging element of epilepsy diagnosis, accurate classification of different epileptic states, is of particular interest and has been extensively investigated. A new deep learning-based classification methodology, namely epileptic EEG signal classification (EESC), is proposed in this paper. This methodology first transforms epileptic EEG signals to power spectrum density energy diagrams (PSDEDs), then applies deep convolutional neural networks (DCNNs) and transfer learning to automatically extract features from the PSDED, and finally classifies four categories of epileptic states (interictal, preictal duration to 30 min, preictal duration to 10 min, and seizure). It outperforms the existing epilepsy classification methods in terms of accuracy and efficiency. For instance, it achieves an average classification accuracy of over 90% in a case study with CHB-MIT epileptic EEG data.

## 1. Introduction

Epilepsy is a chronic disease involving sudden and repeated seizures of brain dysfunction. Due to different starting locations and transmission modes of abnormal electrical activities in brains, there are various complex clinical manifestations of epilepsy, including transient sensory disorders, limb convulsions, loss of consciousness, behavioral disorders, etc. Such clinical manifestations of epilepsy can cause severe physical damage and mental trauma to patients (1). Monitoring electrical activities in the brain and identifying progressing epileptic states and the possible occurrence of seizures can be helpful to mitigate the adverse effects of seizures (2).

The electroencephalogram (EEG) has been a prevalent approach for examining brain activities in epilepsy. For patients with epilepsy, the EEG signals of their brain activities can be categorized into interictal, preictal, and seizure states. When a seizure occurs, the EEG signals exhibit certain unusual patterns. Moreover, the EEG signals of the preictal state and the interictal state also show distinctive patterns. Therefore, these patterns in the EEG signals can be used to differentiate epileptic states, enabling the identification of the progress and the potential occurrence of a seizure and the mitigation of damaging impacts on the patients.

Seizure detection by using EEG signals has been investigated for decades (3–5). For instance, Gotman (6) proposed time-domain feature extraction from the EEG waveform for seizure detection in 1982. In 2006, Jahankhani et al. (7) used a discrete wavelet transform (DWT) to extract EEG features and combined the multilayer perceptron network (MLP) and radial basis function network (RBF) for classification. Wang et al. (8) recognized seizures with different parameters of wavelet coefficients in each frequency band from EEG signals. Acharya et al. (9) decomposed EEG signals into sub-band signals by wavelet packet transform, then took the high-order cumulants of sub-band signals as EEG features, and combined these with a support vector machine (SVM) classifier to complete epilepsy detection. Song et al. (10) used approximate entropy and sample entropy as EEG features, respectively, and combined these with an extreme learning machine (ELM) for automatic detection of epileptic seizures. Based on pattern recognition, a novel method for detecting seizures was presented and tested using the Freiburg database. The method was applied for symbolic data analysis of the EEG signals based on N-gram modeling, a probabilistic pattern recognition technique that identifies the occurrence of symbolic data sequences within data (11). The authors proposed a method based on the mean phase coherence (MPC). MPC was originally proposed by Mormann et al. as a measure of phase synchronization and was found to decrease before seizure onset (12). Williamson et al. proposed a method combining patient-specific machine learning and multivariate features (13). The features were based on the eigenspectra of space delay correlation and covariance matrices computed at multiple time delays.

In recent years, deep learning has started to gain popularity for medical image analysis and bioelectric signal processing. With a large amount of data, it outperforms traditional feature extraction and machine learning methods in pattern detection and image recognition in terms of classification accuracy (14). Deep learning algorithms, especially the convolutional neural network (CNN), are also gradually being adopted for seizure detection. For example, Acharya et al. (15) used a 13-layer depth CNN with EEG signals to detect epileptic seizures and achieved an accuracy of 88.7%. Hu et al. (14) generated a mean amplitude spectrum (MAS) map from EEG signals and incorporated CNN and SVM for feature extraction and classification. The method identified seizure with an accuracy of 86.25%. Besides classification accuracy, sensitivity (i.e., probability of detection) is also used to evaluate the classification performance. Truong et al. (16) applied CNN to learn features from time-frequency energy maps of EEG signals and realized classification with a sensitivity of 89.8%. Khan et al. (17) also used a CNN architecture with six convolutional layers to extract features from the wavelets of EEG signals and achieved seizure detection with an average sensitivity of 87.8%.

However, most of these studies applied domain knowledge to select a specific channel from multichannel EEG signals for analysis, while the data-driven analysis with multichannel epileptic EEG signals remains unexplored. Moreover, there is still room left to further improve the accuracy and efficiency of seizure classification from EEG signals with advanced signal processing and deep learning algorithms. This paper focuses on enhancing the accuracy of classification by analyzing EEG signals of different epileptic states in the brain, which would be helpful for the potential detection of seizures in future study. The epileptic states include an interictal state, a preictal state, and a seizure state. The preictal state can be further divided into two durations: preictal duration to 30 min (denoted as “preictal I”) and preictal duration to 10 min (denoted as “preictal II”). These four categories of epileptic states can be determined from EEG signals. Usually, the small differences in the features of EEG signals between the interictal and preictal states and between the “preictal I” and “preictal II” states are hardly visible or not discernable by eye. However, the dissimilarity in these epileptic states can be captured by deep learning with superior computational power. Therefore, we aim to achieve accurate epileptic state classification by proposing a deep learning-based classification methodology for multichannel EEG signals, named “epileptic EEG signal classification (EESC).” It adopts wavelet transform and power spectrum density (PSD) to preprocess the multichannel EEG signals and incorporates three deep convolutional neural network (DCNN) models for feature extraction and epileptic state classification.

## 2. Epileptic EEG Signal Data

Epileptic EEG signal data in the CHB-MIT database (an open-source public database) are used to verify the effectiveness of the proposed EESC methodology in the case study. Extensive comparisons with the existing epilepsy classification algorithms are implemented. The CHB-MIT database contains child scalp electroencephalogram (sEEG) data from 23 cases (18) that were recorded continuously for 844 h with 163 epileptic seizures. The majority of the sEEG signals are collected through 23 channels with a sampling rate of 256 Hz. Electroencephalographers require EEG abnormalities to persist and evolve for at least 6–10 s before they consider the abnormality to be a seizure, so only the data of patients with seizures of more than 6–10 s were included (16). In our analysis, the EEG signals from 11 patients are used; the seizure duration for these 11 patients are listed in Table 1.

**Table 1:** Seizure duration of eleven selected patients from the CHB-MIT database.

**Patient**	**Gender**	**Age**	**Seizure number**	**Seizure duration (seconds)**
Chb01	F	11	7	402
Chb02	M	11	3	172
Chb03	F	14	7	402
Chb07	F	14.5	3	325
Chb10	M	3	7	447
Chb17	F	12	3	293
Chb18	F	18	6	323
Chb19	F	3	3	236
Chb20	F	8	8	294
Chb21	F	4	4	199
Chb22	F	3	3	294

As mentioned in the Introduction, the EEG signals can be divided into interictal, preictal, and seizure states. Moreover, classifying epileptic seizures 10 to 30 min in advance can help prevent and mitigate the adverse effects of possible seizure occurrence. Up to now, there is no consensus on the earliest detection time before the seizure occurrences. In our analysis, we choose the latest detection time as 10 min, since the EEG signals can show certain peculiar signals when close to the occurrence of seizure (for instance, 5 min before the seizure). It is of use to further divide the preictal state into two durations: preictal duration to 30 min (denoted as “preictal I”) and preictal duration to 10 min (denoted as “preictal II”) for differentiating the importance of duration to the epilepsy progress. Therefore, the EEG signals are divided into four categories: interictal, preictal I, preictal II, and seizure state.

The final dataset for the analysis includes the EEG signals from 11 patients with a total of 56 min of EEG signals in the seizure state and 110 min in the preictal I, preictal II, and interictal states respectively for classification. The EEG signals are divided into frames, with a length of 4 s. Since the EEG signals in the seizure state are shorter than other states, we overlap the consecutive frames of the EEG signals in the seizure state by 2 s. For the classification algorithm, we hold out 70% of the data as the training set and use the remaining 30% as the testing set.

## 3. Classification Methodology

**Figure 1:**
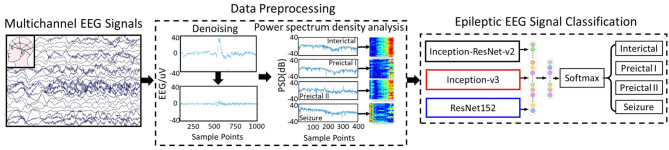
The overall framework of the proposed epileptic EEG signal classification methodology.

In this paper, an epileptic EEG signal classification (EESC) methodology based on deep convolutional neural networks is proposed to classify four critical epileptic states with multichannel EEG signals. The overall framework of the proposed methodology is summarized in Figure 1, and it proceeds in two primary steps: (1) data preprocessing and feature extraction: to denoise multichannel EEG signals and transform them with power spectral density analysis; (2) epileptic EEG signal classification: to classify epileptic states with deep convolutional neural networks (DCNNs) and transfer learning.

### 3.1. Multichannel EEG Signal Preprocessing and Feature Extraction

Multichannel epileptic EEG signals are used to classify four categories of epileptic states, namely the interictal state, preictal I, preictal II, and seizure state. The features of these multichannel EEG signals are represented by characterizing the energy variation of the signals in the frequency domain. To obtain the effective characteristics of multichannel EEG signals, they are first denoised by wavelet transform and then analyzed by power spectrum density (PSD). The two-dimensional images generated from PSD, called power spectrum density energy diagrams (PSDEDs), reflect the energy information of different frequency bands of the EEG signals. PSDEDs are used as features for the subsequent classification since they reveal the differences among the four categories of epileptic states.

#### 3.1.1. EEG Signal Denoising

The original EEG signals are collected on human scalps, so they are inevitably full of noise (such as EEG artifacts, minor interference) and have a low signal-to-noise ratio. In order to reveal the characteristics of EEG signals, they first undergo a denoising procedure (19). For this paper, a wavelet threshold denoising method is used. Particularly, the Daubechies wavelet of order 6 (dB6) is chosen as the mother wavelet for applying discrete wavelet transform (DWT) in denoising (20). The denoised EEG signals are able to highlight the information in different epileptic states, particularly the interictal state, for analysis.

#### 3.1.2. Power Spectrum Density Analysis and Power Spectrum Density Energy Diagram

**Figure 2:**
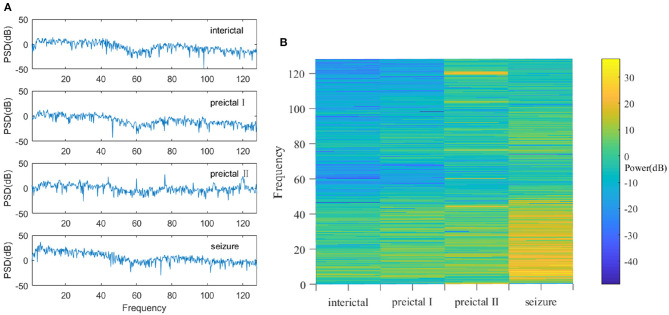
**(A)** The PSD at interictal, preictal I, preictal II, and seizure states. The amplitude of the PSD signal varies greatly among different states. **(B)** Power spectra in interictal, preictal I, preictal II, and seizure states. They are quite different, especially in the low frequency areas (i.e., the region of interest in EEG).

Power spectrum density (PSD) analysis is used on the denoised multichannel EEG signals for feature extraction. The main idea here is to extract the corresponding EEG features by characterizing the energy variation of the signal in the frequency domain (21). PSD can represent the distribution of signal power in the frequency domain (22). As mentioned in the previous section, the EEG signals are segmented into 4-s frames. PSD analysis is implemented on these 4-s frames of the EEG signals, and the resulting periodograms are shown in Figure 2A. It is noticed that the power spectrum density (or energy) of the EEG signals is different among the epileptic seizure, preictal, and interictal states. Therefore, PSD analysis is a viable way to extract features for different epileptic states.

Furthermore, the EEG signals can be transformed into two-dimensional images called power spectrum density energy diagrams (PSDEDs). For instance, when multichannel EEG signals have n channels, the periodogram is obtained for each channel of the EEG signals. If the EEG signals are divided into 32 frequency bands, and PSD functions of different frequency bands can be integrated. A two-dimensional matrix with n rows and 32 columns is then formed and is then normalized to generate a PSDED. The power spectrum densities in the interictal, preictal I, preictal II, and seizure state for one of the patients are shown in Figure 2B. Deep learning will capture such visible differences. Therefore, PSDED is a suitable feature of multichannel EEG signals for the subsequent classification of different epileptic states (23).

### 3.2. Epileptic EEG Signal Classification

**Figure 3:**
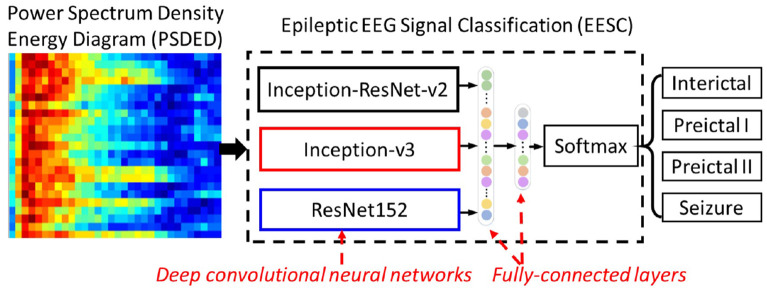
The proposed epileptic EEG signal classification (EESC) integrates three deep convolutional neural networks (DCNNs) and fully connected layers.

Here, epileptic EEG signal classification (EESC) is used for classifying four different epileptic states by using deep convolutional neural networks (DCNNs) and transfer learning with the PSDEDs from the original multichannel EEG signals. The proposed method is shown in Figure 3. It integrates three DCNNs: Inception-ResNet-v2, Inception-v3, and ResNet152. They will be introduced in the following sections. In a transfer learning framework, these three DCNNs are loaded with corresponding pre-trained weights from ImageNet (24). Two fully connected layers and an output classification layer with softmax are concatenated to the DCNNs. The PSDEDs from the multichannel EEG signals are used to train and fine-tune these deep neural networks. Finally, the proposed EESC is ready for classifying the different epileptic states for seizure classification.

#### 3.2.1. Model Structure of the Proposed Epileptic EEG Signal Classification

##### 3.2.1.1. Inception-v3

The architecture of Inception-v3 (25) has been greatly improved on Googlenet. In Inception-v3, the large convolution kernels are decomposed into small convolution kernels to reduce computational complexity and enhance the non-linear expression of features. In the proposed EESC, Inception-v3 has input images with a size of 299 × 299 × 3 and outputs 2,048-dimensional feature vectors.

##### 3.2.1.2. ResNet152

ResNet can alleviate the problem of gradient vanishing in the training of DCNN by adjusting the traditional network structure. Its key structure is to propose the basic network unit, the residual block, by adding a shortcut connection. Residual blocks are used in the whole network as the basic units of ResNet as

(1) $$\begin{array}{r} \;y=f\left(x,w\right)+x \end{array}$$

where *x*,*y*,*f*(*x, w*) represent the input, output, and residual mapping of the block, respectively. By transforming the output y into the residual *f*(*x, w*), the network is more sensitive to the small fluctuations between output y and input x than a plain network structure like VGGNet. In the proposed EESC, ResNet152 transforms the input image with a size of 224 × 224 × 3 into a feature vector of 2,048-dimensions.

##### 3.2.1.3. Inception-ResNet-v2

Inception-ResNet-v2 (26) combines the advantages of the Inception network and ResNet. The residual block is applied to the Inception block, which greatly improves performance and especially accelerates the convergence speed. Such improvement makes the deep network easier to train. In the proposed EESC, Inception-ResNet-v2 transforms the input image with a size of 299 × 299 × 3 into a 1,536-dimensional feature vector.

For each image, the feature vectors extracted from three network features are concatenated into a 5,632-dimensional feature vector. Two fully connected layers with 1,024 and 512 neurons are added to reduce the dimensions, and a dropout layer (0.5) is set behind each fully connected layer to prevent over-fitting. The softmax of the output layer is expressed as:

(2) $$\begin{array}{r} \;P\left(S_{i}\right)=\frac{e^{g_{i}}}{\overset{n}{\underset{k}{\sum }}e^{g_{i}}} \end{array}$$

where *k* represents the number of categories, *i* represents a category in *k*, *g*_*i*_ represents the calculated value of that category, and softmax converts the calculated values into the output probability for each category.

#### 3.2.2. Training Procedure of the Proposed Epileptic EEG Signal Classification

##### 3.2.2.1. Transfer learning

Training the three DCNNs in the proposed EESC requires large amounts of data and time. Transfer learning (27) can be used to optimize network initialization by loading pre-trained weights. It inherits the trained network characteristics and increases training efficiency. There are two ways to apply transfer learning for training classification networks (28): (1) Loading the pre-trained weight, freezing the parameters before the fully connected layer, and only training the fully connected layer. (2) Loading pre-trained weights, and updating the parameters of the whole network during training.

When the current datasets differ greatly from the datasets used in pre-trained weight training, the second approach above is usually adopted. Transfer learning with the pre-training model facilitates the training of classification networks and enables a superior fine-tuning effect. First, the pre-trained weights of ImageNet are loaded to the proposed EESC deep neural networks, and then the weights are updated by the PSDED images from the original multichannel EEG signals. The PSDED images share similar basic features, such as lines, edges, etc., with images from ImageNet. Therefore, transfer learning can still learn important information on the weights of the EECS networks from networks trained with ImageNet data. For the classification algorithm, we hold out 70% of the data as the training set and use the remaining 30% as the testing set.

##### 3.2.2.2. Loss function for EESC

The cross-entropy loss function is widely used in classification problems. Its formula is shown in Equation (3).

(3) $$\begin{array}{r} \;L_{i}=-\left[y_{i}log{ŷ}_{i}+\left(1-y_{i}\right)log\left(1-{ŷ}_{i}\right)\right] \end{array}$$

(4) $$\begin{array}{r} \;L_{batch}=\frac{-\overset{n}{\underset{i}{\sum }}L_{i}}{n} \end{array}$$

where *y*_*i*_ is the label and ŷ_*i*_ is the predicted probability. During training, samples of a batch are fed to the network each time, and the mean value of the loss of samples in the batch is considered as the loss of the batch. Despite its simplicity, it cannot differentiate the losses from different samples in a batch during training and further improve the training accuracy.

An online hard example mining (OHEM) (29) loss function is used to replace the commonly used cross-entropy loss function; its expression is as Equation (5). In OHEM, the loss of a batch sample is sorted in descending order, and the largest k (*top*_*k*_) values are averaged as the final loss. It prioritizes ambiguous samples with large loss values during training and improves the classification accuracy on those.

(5) $$\begin{array}{r} \;L_{ohem}=\frac{-\overset{top_{k}}{\underset{i}{\sum }}L_{i}}{top_{k}} \end{array}$$

## 4. Results and Analysis

### 4.1. Evaluation Metrics

Accuracy, sensitivity, and specificity are the metrics most widely used in the literature for evaluating model performance. They are derived from the correctness of prediction, including true positive (TP: correctly predicts the positive class), true negative (TN: correctly predicts the negative class), false positive (FP: incorrectly predicts the negative class as positive), and false negative (FN: incorrectly predicts the positive class as negative). They can be calculated as follows:

(6) $$\begin{array}{r} \;accuracy=\frac{TP+TN}{TP+TN+FP+FN} \end{array}$$

(7) $$\begin{array}{r} \;sensitivity=\frac{TP}{TP+FN} \end{array}$$

(8) $$\begin{array}{r} \;specificity=\frac{TN}{TN+FP} \end{array}$$

Besides, the confusion matrix is a systematic way to illustrate the classification accuracy for the four categories of epileptic states in the case study. The classification rate displayed in the diagonal of the confusion matrix represents the accuracy of each category, while other values represent the percentage of misclassified samples. This paper focuses on the analysis of four epileptic states in EEG signals of patients to classify the four epileptic states accurately. We can classify the preictal, interictal, and seizure states, and this could potentially help detection.

### 4.2. Performance of the Proposed EESC Methodology

**Figure 4:**
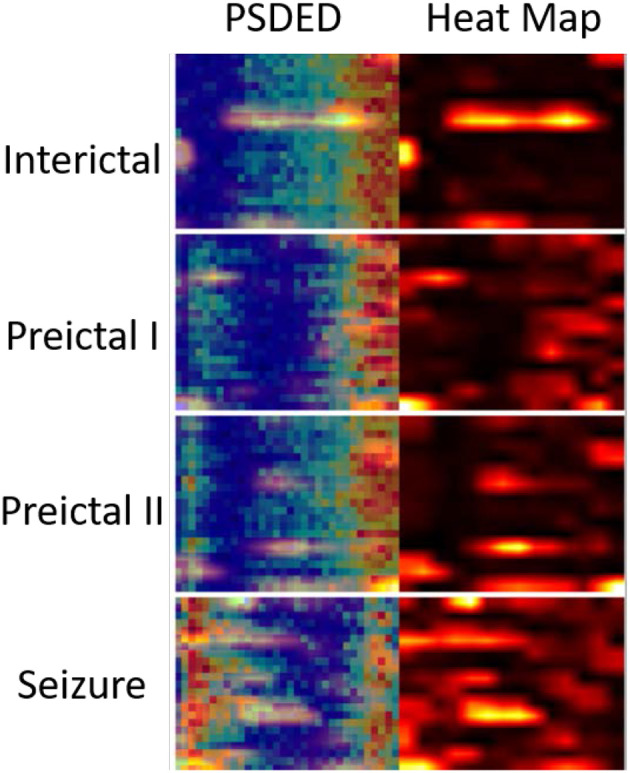
PSDED and its heat map are juxtaposed together for different epileptic states. The heat maps highlight certain important EEG channels for seizure detection.

**Figure 5:**
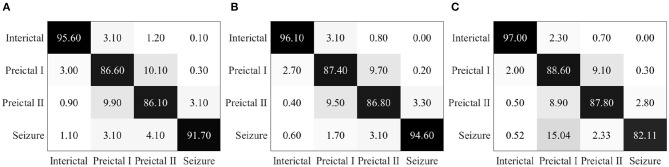
**(A)** Confusion matrix for seizure prediction by using ResNet152 and PSDED. **(B)** Confusion matrix for seizure prediction by using Inception-v3 and PSDED. **(C)** Confusion matrix for seizure prediction by using Inception-Resnet-v2 and PSDED.

Epileptic EEG signals are first used by the three individual deep convolutional neural networks (ResNet152, Inception-v3, and Inception-ResNet-v2) for epileptic state classification. Four states can be classified and heat maps can be generated, as shown in Figure 4. The confusion matrices of their individual performance are shown in Figures 5A–C, respectively. It can be seen that: (1) the Inception-ResNet-v2 model outperforms the other two models except for preictal states; (2) all three models have higher classification accuracy for the interictal state and seizure state than for preictal I and preictal II.

**Figure 6:**
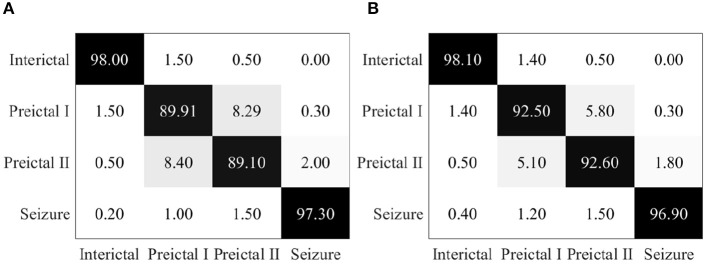
**(A)** Confusion matrix for seizure prediction by using the proposed EESC. **(B)** Confusion matrix for seizure prediction by using the proposed EESC and the OHEM loss function.

Epileptic EEG signals are then used by the proposed EESC methodology. The confusion matrix of EESC is shown in Figure 6A. Compared with the three individual DCNN models above, the classification accuracy of all the four epileptic states is improved in the proposed EESC methodology, but preictal I and preictal II are still not classified as accurately as the other two states.

When integrating the OHEM loss function mentioned in section 3.2 into the EESC methodology, the classification accuracy of preictal I and preictal II is increased by 3 and 4%, respectively, as shown in Figure 6B. The improvement can be attributed to the strength of the OHEM loss function, which prioritizes samples with large losses during training and therefore increases the classification performance of the EESC methodology.

**Figure 7:**
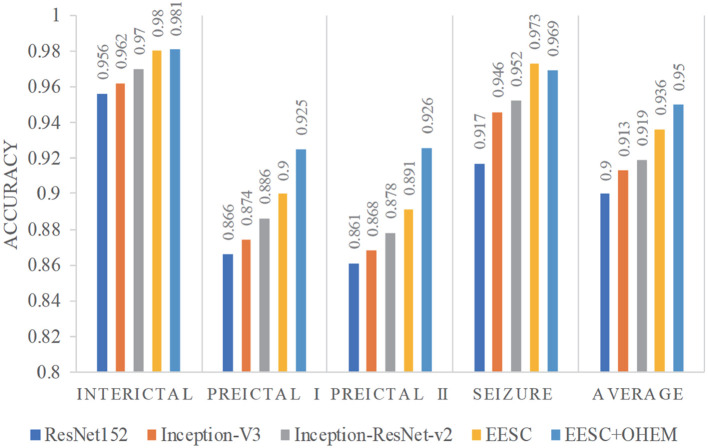
Accuracy of different models for epileptic EEG signal classification.

We summarize the classification accuracy of all the aforementioned models for epileptic EEG signal classification in Figure 7. It is shown that: (1) All three individual DCNNs models have decent classification accuracy, yet the proposed EESC methodology performs even better. (2) The integration of the EESC methodology with the OHEM loss function has superior performance in seizure classification. (3) For all models, the classification accuracy of the seizure state and interictal state is higher than that of preictal I and preictal II.

**Figure 8:**
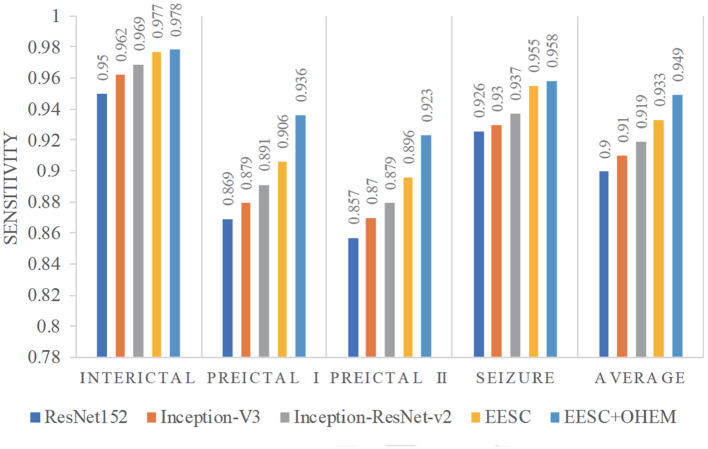
Sensitivity of different models for epileptic EEG signal classification.

**Figure 9:**
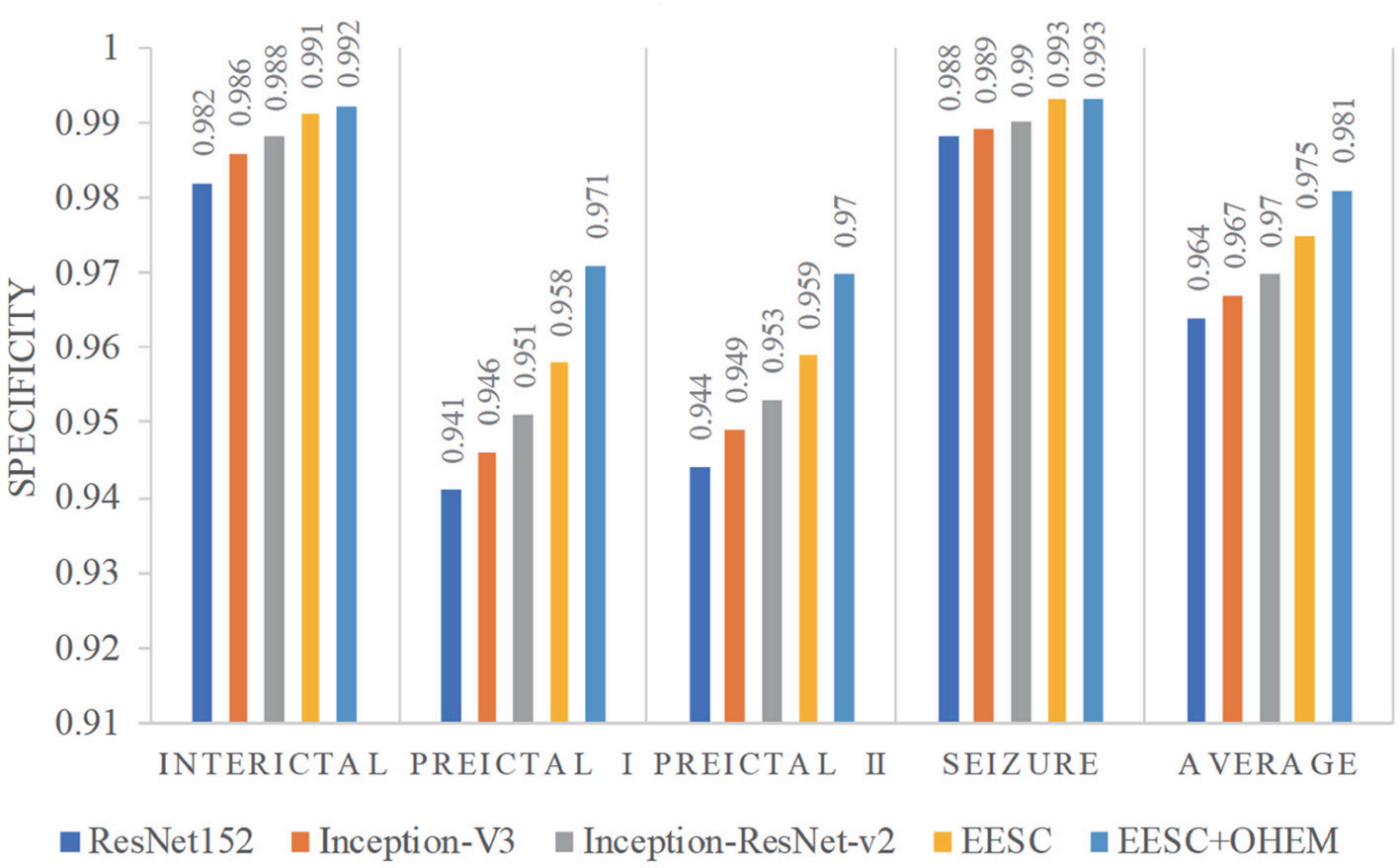
Specificity of different models for epileptic EEG signal classification.

Furthermore, we compare the classification results from the aforementioned models in terms of sensitivity and specificity. In this case study, sensitivity is the percentage of correct classification of a particular epileptic state, while specificity is the percentage of correct classification for other epileptic states. They are summarized in Figures 8, 9, respectively. The proposed EESC method with an OHEM loss function outperforms other methods in terms of both sensitivity and specificity. It has high sensitivity (97.8, 93.6, 92.3, and 95.8%) and specificity (99.2, 97.1, 97, and 99.3%) in classifying the four epileptic states (interictal, preictal I, preictal II, and seizure).

Finally, state-of-the-art research in epileptic EEG signal classification is applied to the dataset from the CHB-MIT database to implement a comparison with the proposed EESC methodology. The results are summarized in Table 2. It is noted that the proposed EESC methodology outperforms other methods in terms of classification accuracy on preictal duration (31) in epileptic EEG signals.

**Table 2:** Comparison with the state of the art in the literature for the preictal duration of EEG signal classification.

**Authors**	**Feature**	**Classifier**	**Accuracy**	**Sensitivity**	**Specificity**	**Preictal duration (min)**
Truong et al. (16)	STFT spectral images	CNN	–	89.1	–	5
Chu et al. (30)	Phase locking value	SVM	–	82.44	82.76	5
Khan et al. (17)	Wavelet transform coefficients	CNN	–	83.33	–	10
Hu et al. (14)	MAS	CNN	75.28 73.29	–	–	20 40
Our proposed work	PSDED	EESC	92.6 92.5	92.3 92.6	97 97.1	10 30

## 5. Discussion

### 5.1. Effective EEG Features Represented by PSDED

The PSDED obtained from PSD analysis can represent the energy level at each frequency of the epilepsy EEG signals. It is noted from Figures 2B, 4 that compared to the other three epileptic states, the PSDED of the seizure state has an increasing energy level at low frequencies but a decreasing energy level at high frequencies. In contrast, the PSDED of the interictal state has a high energy level at high frequencies but a low energy level at low frequencies. Since EEG operates at low and medium frequencies, it can better capture the seizure occurrence at low frequencies with high energy level. Moreover, the analysis of PSDEDs in preictal I and preictal II shown in Figures 2B, 4 demonstrates that there are many similarities between these two states. Both PSDEDs show a high energy level at high frequencies and a low energy level at low frequencies. The subtle differences in the PSDEDs of these two states can be effectively classified by features extracted by DCNNs, as shown in Figure 4.

The heat map of PSDEDs in Figure 4 is an effective illustration of differences between the four epileptic states. It is obtained through the training of DCNNs and can highlight the unique features in these four categories. It is a useful indication of seizure occurrence. The increasing energy level at low frequencies in the heat maps indicates a looming seizure occurrence. The heat maps in Figure 4 also highlight certain EEG channels such as T8-P8, F3-C3, and FP2-F4, which are verified to be important EEG channels for seizure detection (32, 33). Therefore, the PSDED is once again proved to be an appropriate and effective method to extract EEG signal features for seizure classification.

### 5.2. Comparison Between Conventional Models and the Proposed EESC Methodology

Essentially, this proposed EESC methodology is a multi-classification algorithm, mainly for four categories of epileptic EEG classification. The purpose of this study is to accurately classify EEG signals of different states.

Conventional models for epileptic seizure classification use wavelet transform (WT), short-time Fourier (STF), and other methods to extract features from EEG signals and then use machine learning to classify them. For comparison, three popular machine learning algorithms, i.e., support vector machine (SVM) (34), extreme learning machine (ELM) (35), and linear discriminant analysis (LDA) (36) are used as benchmark models for the classification of different EEG states. The EEG signals are decomposed by wavelet transform, and the reconstructed wavelet coefficients are used as features to classify the epileptic states with these selected machine learning algorithms.

**Figure 10:**
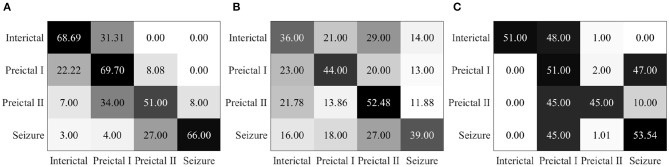
**(A)** Confusion matrix for seizure prediction by using wavelet transform and SVM. **(B)** Confusion matrix for seizure prediction by using wavelet transform and ELM. **(C)** Confusion matrix for seizure prediction by using wavelet transform and LDA.

The results of these conventional models with different machine learning algorithms are summarized in confusion matrices in Figure 10. The classification rates presented in the diagonal represent the correct accuracy of each category. It is noted that the classification accuracy of conventional methods with SVM, ELM, and LDA is only 63.85, 42.8, and 50.14%, respectively. Compared with the performance of the proposed EESC methodology shown in Figure 7, they underperform significantly. The reason for poor classification performance could be that wavelet transform does not preserve some important information in the features from the EEG signals, and traditional machine learning algorithms are not sensitive enough to discover the patterns in the weak features.

**Figure 11:**
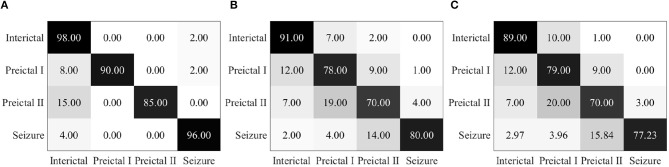
**(A)** Confusion matrix for seizure prediction by using DCNNs and SVM. **(B)** Confusion matrix for seizure prediction by using DCNNs and ELM. **(C)** Confusion matrix for seizure prediction by using DCNNs and LDA.

We augment the feature extraction in those conventional models with the proposed ensemble of DCNNs on the power spectrum density energy diagram (PSDED) obtained from original EEG signals. With the PSDED images, the three machine learning classifiers (SVM, ELM, and LDA) are still used to classify epileptic states. The performance metrics obtained are represented by the confusion matrices in Figure 11. It is found that the classification performance is improved by using features extracted by DCNNs from PSDEDs. In this case, the accuracy of conventional methods with SVM, ELM, and LDA are 92.25, 79.75, and 61.30%, respectively. It can be inferred from the comparison that DCNNs can extract more informative features from PSDED to increase classification accuracy for these traditional machine learning algorithms.

Furthermore, by comparing the classification results with the proposed EESC methodology in Figure 6, we can conclude the proposed EESC methodology has significantly better performance in classifying epileptic EEG signals than the conventional methods with machine learning classifiers. Through confusion matrices, we can see that the challenges in classification are mainly in preictal I and preictal II. This is usually due to the similarity between the EEG signal features of the preictal states. With DCNNs, the proposed EESC methodology can learn the subtle differences in the features of EEG signals, enabling better differentiation between preictal I and preictal II.

### 5.3. Performance Evaluation for the Proposed EESC Methodology

Accuracy, sensitivity, and specificity are used in this paper to evaluate the performance of the classification of epileptic states. For instance, as illustrated in Table 1, the proposed EESC methodology integrating the OHEM loss function can achieve 92.6% sensitivity and 97.1% specificity in classification. This means that the methodology has 92.6% correct seizure classification and 97.1% correct non-seizure classification. On the other hand, it means we fail to detect 7.4% of the seizure occurrences and make 2.9% false classification of seizure occurrence. Here, we discuss the potential impacts of the missed classification and false classification.

## 6. Conclusions

Accurate classification could potentially reduce damage caused by seizure occurrence. In this paper, we propose a novel epileptic EEG signal classification (EESC) methodology using DCNNs based on transfer learning and the power spectrum density energy diagrams (PSDED) to classify different epileptic states (i.e., interictal, preictal I, preictal II, and seizure). The methodology is verified by the multichannel EEG signals in the CHB-MIT database. It can be concluded through the study that (1) the proposed EESC methodology outperforms other benchmark models in classifying different epileptic states; (2) DCNNs have excellent feature extraction ability from the power spectrum density energy diagram (PSDED) of multichannel EEG signals; (3) the model trained with an OHEM loss function prioritizes samples with large loss and achieves high classification accuracy. In medical practice, the proposed EESC methodology could have important practical impacts on epilepsy diagnosis and treatment. For instance, to patients, the high classification accuracy of preictal states (i.e., preictal I, preictal II) of EESC can enable reliable and timely warning; to doctors, the high classification accuracy of EESC can facilitate their understanding of the categories of epilepsy in patients, enabling effective epilepsy prevention and treatment.

Consequently, this work addresses one of the significant challenges for accurate epileptic state classification with multichannel EEG signals. As part of our future research, we aim to improve the EESC methodology in the following ways in order to better serve epilepsy prevention and treatment: (1) to design precise tags for EEG signals in the preictal state to further improve the classification performance; (2) to utilize the proposed classification of EEG to detect and/or predict seizures; (3) to further reduce the false detection of seizure occurrence, for instance, by incorporating temporal correlation among frames of EEG signals; (4) to enable the diagnosis of different categories of epilepsy by locating the focus of epileptic seizures.

## Data Availability Statement

Publicly available datasets were analyzed in this study. This data can be found here: http://physionet.mit.edu/physiobank/database/chbmit/.

## Author Contributions

YG: substantial contributions to the conception or design of the work. YZ: provide approval for publication of the content. JL: revising it critically for important intellectual content. QC and BG: analysis and interpretation of data for the work.

## Conflict of Interest

The authors declare that the research was conducted in the absence of any commercial or financial relationships that could be construed as a potential conflict of interest.
